# Genotype to Phenotype: CRISPR Gene Editing Reveals Genetic Compensation as a Mechanism for Phenotypic Disjunction of Morphants and Mutants

**DOI:** 10.3390/ijms22073472

**Published:** 2021-03-27

**Authors:** Cristy M. Salanga, Matthew C. Salanga

**Affiliations:** 1 Office of the Vice President for Research, Northern Arizona University, Flagstaff, AZ 86011, USA; cristy.salanga@nau.edu; 2 Department of Biological Sciences, Northern Arizona University, Flagstaff, AZ 86011, USA

**Keywords:** genetic plasticity, genetic compensation, reverse genetics, zebrafish, CRISPR/Cas, transcription activator-like effector nucleases (TALENs), morpholino, nonsense-mediated mRNA decay

## Abstract

Forward genetic screens have shown the consequences of deleterious mutations; however, they are best suited for model organisms with fast reproductive rates and large broods. Furthermore, investigators must faithfully identify changes in phenotype, even if subtle, to realize the full benefit of the screen. Reverse genetic approaches also probe genotype to phenotype relationships, except that the genetic targets are predefined. Until recently, reverse genetic approaches relied on non-genomic gene silencing or the relatively inefficient, homology-dependent gene targeting for loss-of-function generation. Fortunately, the flexibility and simplicity of the clustered regularly interspaced short palindromic repeats (CRISPR)/Cas system has revolutionized reverse genetics, allowing for the precise mutagenesis of virtually any gene in any organism at will. The successful integration of insertions/deletions (INDELs) and nonsense mutations that would, at face value, produce the expected loss-of-function phenotype, have been shown to have little to no effect, even if other methods of gene silencing demonstrate robust loss-of-function consequences. The disjunction between outcomes has raised important questions about our understanding of genotype to phenotype and highlights the capacity for compensation in the central dogma. This review describes recent studies in which genomic compensation appears to be at play, discusses the possible compensation mechanisms, and considers elements important for robust gene loss-of-function studies.

## 1. Introduction

Forward genetic screens provided the foundational studies to dissect biological pathways. However, reverse genetic approaches created the ability to interrogate the function of a single gene of interest, delivering key insights and a deeper understanding of complex gene networks and individual gene functions. Although reverse genetic techniques have been utilized in a wide variety of organisms, including mouse (reviewed in [1,2]), *Arabidopsis* (reviewed in [3,4]), *Xenopus* [5,6,7], and *Drosophila* (reviewed in [8,9]), among others, this review primarily addresses recent studies performed in zebrafish that highlight genetic compensation given the breadth of literature relating to this model organism.

## 2. Genetic Knockdown

Disrupting a particular gene was initially accomplished via gene knockdown and then with the invention of a new series of techniques by gene knockout. Knockdown methods have largely included antisense oligonucleotides (reviewed in [10]), morpholinos [11], and RNAi [12]. Antisense oligonucleotides pair with their mRNA complement and induce degradation through the cleaving action of RNAse H [11]. Similarly, morpholinos are modified, synthetic antisense oligonucleotides comprising a morpholine ring which complementarily bind the mRNA of interest and inhibit translation via two main methods of action: ribosomal blocking or interference with precursor mRNA splicing. RNAi mediates gene knockdown via the modulation of the RNAi pathway using a series of small RNA effector molecules, including endogenous microRNAs (miRNAs) or small interfering RNAs (siRNAs) which are evolutionarily conserved to regulate mRNA expression by either causing their degradation or preventing translation [12].

In particular, morpholino antisense oligonucleotides have provided countless advancements as an essential knockdown tool to understand hundreds of genes of interest; however, their off-target and toxicity effects have been well documented and have opened debate as to the validity of their induced phenotypes [13,14,15,16]. Specifically, morpholinos have been observed to non-specifically induce the *p*53-dependent apoptosis pathway, confounding the morphant’s phenotypic outcome [17]. Circumventing this off-target effect with *p53* knockdown by a morpholino targeting *p53* itself may alleviate these issues and provide validity to the observed phenotype [17]. However, it is worth noting that *p53* is involved in multiple biological processes, and CRISPR-induced *p53* mutants display developmental and behavioral abnormalities [18]. Thus, careful analysis is still needed in phenotype characterization with *p*53 inhibition. Additionally, morpholinos can activate interferon-stimulated genes (ISGs) *isg15* and *isg20*, apoptosis pathway gene *casp8*, as well as several other genes related to cellular stress pathways, including *phlda3*, *mdm2*, and *gadd45aa* in zebrafish [19].

## 3. Morphant and Mutant Disjunction

As the generation of site-specific mutations has become mainstream in research, more studies have uncovered phenotypic discrepancies between morphants and mutants. Generating over twenty mutant lines of vasculature-related genes, Kok et al. observed that ten mutants failed to show the expected phenotypes and only three mutant lines (*gata2a*, *ccbe1*, and *flt4)* displayed similar lymphatic defects to the previously characterized morphants [14,20,21]. In genes found to be required for intersegmental vessel development (*amot*, *elmo1*, *ets1*, *fmnl3, nrpl1a, pdgfrb*), all mutants displayed normal morphology, contradicting the published phenotypes [22,23,24,25,26,27]. Considering the entirety of their results, Kok et al. suggested that off-target effects were more prevalent than previously anticipated, a view shared by others [14,28]. Interestingly, the group noted that the genetic mutations were expected to create truncated proteins to result in nonsense-mediated mRNA decay [14].

Inconsistencies in observed phenotypes between morphants and mutants continue to be reported, which have led to conclusions of the gene of interest being dispensable in development. Law and Sargent, using the TALEN-based knockout of serine/threonine protein kinase *pak4* in zebrafish, found that the morphology of the *pak4*-null mutant was normal with none of the lethal vascular outcomes reported in both the null mouse line and the zebrafish morphants [29,30]. The authors generated three mutants comprising two- or four-base pair (bp) deletions which resulted in frameshifts and premature termination codons (PTCs). Based on additional analysis, the authors conclude that *pak4* is dispensable for zebrafish development. In another study, Moreno et al. investigated the TALEN knockout of *islet2a* in zebrafish where previous morpholino studies presented the disruption of motor neuron axon morphology with truncated axons; *islet2a* homozygous mutants, comprising a 13-bp deletion leading to a PTC, displayed normal morphology [31]. Additionally, Place and Smith found that the zebrafish mutant of bHLH transcription factor *atoh8* also failed to phenocopy the reported morpholino-induced developmental defects in pancreatic and endothelial cell differentiation and regulation; such results led the authors to conclude that *atoh8* is dispensable in zebrafish development [32]. Perhaps the genes in these examples are in fact dispensable but taking into consideration the new evidence surfacing for genetic compensation, additional studies may provide fascinating insights in their role during development and central dogma durability.

In many cases, morpholino off-target effects are responsible for the observed differences [28]; however, as previously mentioned, more data implicate other less-described mechanisms at play. Herein, we will discuss the recent literature supporting genomic plasticity and a transcriptional adaptation referred to as genetic compensation. Table 1 provides a summary of findings.

## 4. Plasticity in the Central Dogma of Molecular Biology

In 1953, the structure of DNA was solved [41,42,43,44,45], providing context to Thomas Hunt Morgan’s chromosomal theory of inheritance [46] and paving the way to the central dogma of molecular biology: the concept that genetic sequence information is stored in the form of DNA, which is transcribed to RNA, and then translated into protein [47]. According to the central dogma, many defects (i.e., mutations) in DNA sequence can be carried over into mRNA which, depending on the nature of the sequence change, affect mRNA translation to protein. Generally, forward genetic screens have supported the central dogma and have suggested little tolerance for mutations in coding sequence. Fast forwarding five decades, there is evidence for DNA transcription to RNA that avoids genomic mutations through a plurality of mechanisms including the usage of alternative splicing, alternative transcriptional initiation, and alternative termination sites. In other cases, mRNA containing the mutations coded in the DNA retain translatability, possibly through ribosomal read-through and alternative translational start sites.

## 5. Mechanisms for Genetic Compensation

It is understood that genetic robustness is a key survival feature, allowing organisms to persist through harmful mutations and other perturbations. Many studies have proposed mechanisms for describing conventional genetic robustness, including redundant genes [48], regulatory cellular networks [49], and adaptive mutations as seen in yeast [50]; additional reviews can be found in [51,52,53,54]. In view of the morphant/mutant phenotypic discrepancies that have been largely thought to be contributed to by morpholino off-target effects, multiple publications provide evidence of an alternative explanation which supports the concept of genetic durability, commonly referred to as genetic compensation; however, the underlying mechanisms have yet to be completely characterized although these recent studies further provide substantial elucidation.

The discrepancies between morphants and mutants have led to inquiries as to the plasticity of the central dogma and a search for alternate explanations including transcriptional adaptation and genetic compensation. Studies by Rossi et al. investigated if these differences were in fact due to toxic off-target effects by analyzing the EGF-like-domain multiple 7 (*egfl7*) TALEN-induced mutants and the respective morphant [33]. *Egfl7* is an endothelial cell-derived secreted factor associated with cellular proliferation and migration during vascular development. In zebrafish, morphants demonstrated major impairment of angiogenesis and vascular tubulogenesis [55]. Despite the morphant phenotype, Rossi et al. found almost no defects in *egfl7* mutants as well as mutants co-injected with the *egfl7* morpholino, suggesting that off-target effects were not at play for the resulting outcome. Instead, several other genes in the Emilin protein family, specifically *emilin3a*, were upregulated which share a functional domain with *egfl7*, leading to the question of whether genetic compensation could be occurring [33].

El-Brolosy and Stainier summarized additional genes whose discrepancies have been documented between morphants and mutants [56]. Additionally, their publication reviewed proposed models and potential triggers for transcriptional adaptation which include the DNA damage response and mutations initiating mRNA degradation through known mRNA surveillance pathways. One such mRNA surveillance pathway recognized as nonsense-mediated mRNA decay (NMD) represents a conserved mechanism to detect defective or incorrect messages comprising PTCs which may create non-functioning proteins. Studies stemming back to the late 1970s in yeast demonstrated that a PTC mutation in the *ura3* gene reduced mutant mRNA levels while not affecting the rate of the mRNA synthesis [57]. More recent studies have elucidated this mechanism’s characteristics beyond a simple mRNA quality control method to implicate a role in the post-transcriptional gene regulation of mRNAs with specific features which make them physiologically sensitive to NMD with a PTC [58]. The complexity of this mechanism is beyond the scope of this article but is described in detail in the reviews [59,60,61,62].

In a subsequent article, the Stanier research group revealed more details on a potential acting mechanism through the transcriptomic analysis of zebrafish mutant alleles comprising PTCs and mutant mRNA decay [34]; such genes included *hbegfa*, *vcla*, *hif1ab*, *vegfaa*, *egfl7*, and *alcama*, all of which showed the upregulated expression of a compensating related family member or paralogue *hbegfb*, *vclb*, *epas1a* and *epas1b*, *vegfab*, *emilin3a*, and *alcamb,* respectively. Additionally, El-Brolosy et al. demonstrated that injecting the wild-type mRNA of these genes into their respective mutant resulted in no effect on the compensation response (i.e., no upregulation of the related genes), implying that the mechanism needed to be triggered upstream of protein loss. The reasoning was that if mutant mRNA degradation is the trigger for this compensation mechanism, mutant alleles that were unable to produce an mRNA transcript would not induce this response. The authors deleted either the promoter region or entire gene locus via CRISPR/Cas9 editing and demonstrated that such mutations in *hbegfa*, *vegfaa*, and *alcama* did not upregulate their respective compensating gene, thus indicating that mutant mRNA decay was necessary to induce the genetic compensation. Furthermore, a mutation in the nonsense-mediated mRNA decay pathway essential factor *upf1* in the *hbegfa*, *vegfaa*, and *vcla* mutants resulted in less mutant mRNA decay and no genetic compensation [34].

Further confirming the observations relating to compensation through mRNA decay, Ma et al. also found that mutant mRNAs comprising a PTC prompt what the authors refer to as the genetic compensation response (GCR) [35]. This group investigated the zebrafish mutant phenotype for *capn3a*, an intracellular calcium-dependent cysteine protease expressed in the brain and liver bud during early development, of which the morphant outcome produced a small liver. Using TALENs to delete a 14-bp segment in exon 1 resulting in a PTC, homozygous mutants produced normal liver morphology but also showed the upregulation of 10 *capn* family member transcripts relative to wild-type and morphants, with the highest upregulation being of *capn8* and *capn12*.

Perhaps most interesting to dissecting this compensation mechanism was that Ma et al. demonstrated that increased histone H3 Lys4 trimethylation (H3K4me3) was present at transcription start sites (TSS) of the upregulated *capn* family members, particularly *capn8* and *capn12*, in mutants versus wild-types. Furthermore, the morpholino knockdown of nonsense-mediated mRNA decay essential factor *upf3a* significantly reduced the presence of H3K4me3 at the *capn8* and *capn12* TSS regions in mutants but not in wild-types [35]. Thus, this suggests that this increase in H3K4me3 presence at the TSS regions of genes implicated in the compensation of *capn3* is dependent on *upf3a* and the nonsense-mediated mRNA decay pathway.

The authors further detail a proposed mechanism for the GCR, identifying that components of the Complex Proteins Associated with Set1 (COMPASS) Complex (reviewed in [63]), specifically *wdr5*, directly interact with *upf3a*; the morpholino knockdown of *wdr5* in *capn3a* recapitulated the small liver phenotype initially observed in *capn3a* morphants as well as showed down the regulation of compensating genes *capn8* and *capn12*, implying that GCR was inhibited by *wdr5* depletion. Taken together, Ma et al. provides the scaffold of a mechanism in which mutations resulting in mRNAs with PTCs initiate nonsense-mediated mRNA decay and upregulate compensatory genes through COMPASS Complex elements associated with H3K4me3 at the TSS regions of these related genes. Additional discussion, research progress, and a detailed proposed mechanism of the GCR is reviewed in [64].

Sztal and Stainer reviewed the genetic compensation response and posed the question of if this mechanism is conserved across species [65]. Although much of the research discussed herein has employed zebrafish to understand this transcriptional adaptation, several other model organisms, including mouse [66,67,68], *Drosophila* [69,70,71], yeast [72,73,74], and *Arabidopsis* [75,76,77,78], report similar discrepancies which would benefit from in-depth compensation analyses. Additionally, this compensation phenomenon was also evidenced in *Caenorhabditis elegans*, which also involved nonsense-mediated mRNA decay among other important factors of Argonaute proteins and Dicer [79].

## 6. Gene Compensation Examples

Several examples of genetic compensation have been recently documented. Salanga et al. reported notable discrepancies between knockdown and knockout studies of zebrafish pregnane X receptor (*pxr; nr112*), a nuclear receptor and zinc finger transcription factor involved in transcriptional responses to xenobiotic exposure with conserved functions across various species [36,80]. Among other functions, Pxr, upon ligand binding, induces the expression of the xenobiotic-metabolizing enzyme cytochrome P450 3A (Cyp3a). Early genetic knockout studies of *pxr* in mouse demonstrated the loss of *pxr* target gene *cyp3a* expression in response to known activators dexamethasone and pregnenolone-16 alpha-carbonitrile [81]. Later studies in zebrafish using morpholino knockdown against *pxr* also predictably led to the loss of transcriptional activation of *cyp3a65* [80]. Using CRISPR/Cas9 gene editing, Salanga et al. generated two *pxr* mutant lines: one with a 108-bp deletion in the DNA-binding domain of exon 2 leading to a frameshift and the other a 236- bp deletion, that includes a 37-bp deletion in exon 7 and near total deletion of intron 7 and exon 8. The sequence of the expressed transcript from the exon 7, 8 mutant allele revealed the direct splicing of exons 6–9. Notably, PTCs were identified in coding strand DNA; unexpectedly, both mutants retained the wild-type-like activation of *cyp3a65* in response to *pxr* ligand, pregnenolone suggesting the expression of functional gene product [36]. Although this study did not investigate the possibility of compensatory mechanisms of *pxr*, this pathway should be investigated for genetic compensation considering the conserved evolution of *pxr* across species.

Diofano et al. investigated the consequences of CRISPR/Cas9-induced zebrafish knockout of co-chaperone Bcl2-associated athanogene 3 (*bag3*) wherein the *bag3* morphant has been documented to cause cardiovascular defects including dilated cardiomyopathy and myofibrillar myopathy [37]. The authors found that the *bag3* homozygous mutant had a surprisingly complete and preserved skeletal muscle structure and heart formation despite confirmation of substantially reduced *bag3* mRNA levels. Interestingly, *bag2*, a closely related *bag* family member, was significantly upregulated in the *bag3* mutant, suggesting it is a candidate for gene compensation. The injection of *bag2* splice-blocking morpholino into *bag3* mutant embryos recapitulated the cardiovascular defects witnessed in the *bag3* morphant. Diofano et al. further demonstrated that the inhibition of the nonsense-mediated mRNA decay pathway via NMD essential gene *upf1* knockdown in *bag3* mutants prevented the degradation of mutant *bag3* mRNA, foiling compensation by *bag2* and leading to the expected heart and skeletal muscle defects [37].

Zhu et al. provides another example of deleterious mutations in zebrafish basement membrane glycoprotein Nidogen 1 (*nid1*) leading to genetic compensation by a Nidogen family member *nid2* [38]. The Nidogen family comprises *nid1a*, *nid1b*, *nid2a*, and *nid2b* which share structural similarity but low sequence comparison and localize differently in the basement membrane. The knockdown of *nid1a* by morpholino resulted in a shortened body length [38]. Interestingly, a homozygous mutant of *nid1a* with a frameshift leading to a PTC also led to this same shortened phenotype, but only temporally from 1 to 3 days post-fertilization (dpf); the shortened body phenotype gradually disappeared between 4 to 5 dpf, unlike morphants, which retained the shortened body length. Zhu et al. determined that mutant *nid1a* mRNA was indeed degraded through the nonsense-mediated mRNA decay pathway. Furthermore, the temporary mutant phenotype was found to be compensated by *nid1b* and *nid2a* [38].

She et al. details the compensation of zebrafish erythropoietin a (*epoa*) which has been shown by morpholino-mediated knockdown to be essential in pronephros development among other functions [39]. With *epoa* knockdown, morphants display severe anemia and pronephros defects [40]. However, CRISPR/Cas9-generated homozygous mutants with PTC in exon 2 develop normally, suggesting genetic compensation. Indeed, family member erythropoietin b (*epob*) was upregulated in mutants compared to wild-type zebrafish. Furthermore, the morpholino knockdown of *epob* in *epoa* mutants recapitulated the pronephros defects [39]. An interesting outcome of this study was that while *epoa* mutants displayed normal pronephros development, this effect was temporally limited to larval stages, suggesting only an overall partial compensation as *epoa* and *epob* were shown to express differentially in time.

Considering the new data developing on genetic compensation, it is also important to assess whether genetic compensation is at the core of the observed phenotype. Huang et al. described frameshift mutations leading to wild-type outcomes [82]. In the type I toxin (*ibsC*) and *dinQ* genes in *Escherichia coli*, the authors found that +1 and −1 frameshift mutants retain its normal toxicity and suggest that the proteins encoded by the shifted reading frames encode hidden proteins with the same function. In response to Huang et al., Mankin countered with a simpler, alternative explanation in line with the central dogma [83]; his work showed that the *ibsC* gene indeed starts with two AUG codons wherein a −1 frameshift would lead to the use of the second in-frame start codon and thus not affect the gene’s function. Additionally, the +1 frameshift in the *dinQ* gene resulted in the deletion of the A in the AUG and the generation of an alternative start GUG codon. While it is exciting to imagine scenarios that revolutionarily break the central dogma, there may also be simpler reasons to the observed outcomes.

## 7. Conclusions

Plasticity in the central dogma is an emerging theme from loss-of-function studies in model organisms. The disjunction between morpholino knockdown and genetic knockout phenotypes in zebrafish has been explained as off-target effects; however, in some cases the phenotypic discrepancy can be explained by genetic compensation in which a wild-type phenotype is derived through alternative genetic pathways or transcriptional/translational plasticity. In order to achieve a thorough understanding of a gene’s function, a multipronged approach that includes gene knockdown and genetic mutations should be implemented. Furthermore, the ease CRISPR/Cas9 mutagenesis warrants creation of multiple unique mutants (e.g., with and without PTC).

## Figures and Tables

**Table 1:** Summary of morphant and mutant phenotype disjunction. Dpf: days post-fertilization.

Target Gene	Morphant Phenotypes	Mutant Phenotypes	Compensatory Gene	References
*pak4*	Defects in primitive myelopoiesis, vasculature, and somite development; lethality by 6–7 dpf	Normal primitive myelopoiesis	N/A	[29,30]
*islet2a*	Presumptive motor neurons fail to extend axons required for proper motor function	Normal axon formation and morphology	N/A	[31]
*atoh8*	Defects in body curvature, retinal lamination, and skeletal muscle structure	Normal morphology	N/A	[29]
*egfl7*	Severe defects in vascular development including intersegmental vessel formation, and pericardial edema	No obvious defects	*emilin3a*	[33,34]
*capn3*	Small liver size	Normal liver development	*capn8, capn12*	[35]
*pxr*	Loss of *pxr* transactivation of target genes *cyp3a65* and self-induction of *pxr*	No obvious impairments to *pxr* function or morphology	N/A	[36]
*bag3*	Heart failure due to heart and skeletal muscle structure and function disruption	No obvious heart or skeletal muscle defects	*bag2*	[37]
*nid1*	Retained shortened body length	Delayed body lengthening; restored at 4–5 dpf	*nid1b, nid2a*	[35,38]
*epoa*	Morphological defects in pronephric structures	Normal pronephros development	*epob*	[39,40]

## Data Availability

Not applicable.
